# Understanding mitochondrial DNA maintenance disorders at the single muscle fibre level

**DOI:** 10.1093/nar/gkz472

**Published:** 2019-05-31

**Authors:** Diana Lehmann, Helen A L Tuppen, Georgia E Campbell, Charlotte L Alston, Conor Lawless, Hannah S Rosa, Mariana C Rocha, Amy K Reeve, Thomas J Nicholls, Marcus Deschauer, Stephan Zierz, Robert W Taylor, Doug M Turnbull, Amy E Vincent

**Affiliations:** 1 Department of Neurology, University of Ulm, 89075, Ulm, Germany; 2 Department of Neurology, University of Halle-Wittenberg, 06120, Halle/Saale, Germany; 3 Wellcome Centre for Mitochondrial Research, Institute of Neuroscience, Newcastle University, Newcastle upon Tyne, NE2 4HH, UK; 4 NHS Highly Specialised Mitochondrial Diagnostic Laboratory, Newcastle upon Tyne Hospitals NHS Foundation Trust, Newcastle upon Tyne, NE2 4HH, UK; 5 Centre for Ageing and Vitality, Newcastle University, Newcastle upon Tyne, NE2 4HH, UK; 6 Department of Neurology, Technical University Munich, 81675, Munich, Germany

## Abstract

Clonal expansion of mitochondrial DNA (mtDNA) deletions is an important pathological mechanism in adults with mtDNA maintenance disorders, leading to a mosaic mitochondrial respiratory chain deficiency in skeletal muscle. This study had two aims: (i) to determine if different Mendelian mtDNA maintenance disorders showed similar pattern of mtDNA deletions and respiratory chain deficiency and (ii) to investigate the correlation between the mitochondrial genetic defect and corresponding respiratory chain deficiency. We performed a quantitative analysis of respiratory chain deficiency, at a single cell level, in a cohort of patients with mutations in mtDNA maintenance genes. Using the same tissue section, we performed laser microdissection and single cell genetic analysis to investigate the relationship between mtDNA deletion characteristics and the respiratory chain deficiency. The pattern of respiratory chain deficiency is similar with different genetic defects. We demonstrate a clear correlation between the level of mtDNA deletion and extent of respiratory chain deficiency within a single cell. Long-range and single molecule PCR shows the presence of multiple mtDNA deletions in approximately one-third of all muscle fibres. We did not detect evidence of a replicative advantage for smaller mtDNA molecules in the majority of fibres, but further analysis is needed to provide conclusive evidence.

## INTRODUCTION

Multiple mitochondrial DNA (mtDNA) deletions are found in mitochondrial disease patients with recessive or dominant pathogenic variants in proteins required for mtDNA replication and maintenance (1–4). In patients with mtDNA maintenance disorders, the multiple mtDNA deletions accumulate later in life; however, some gene variants are also associated with symptoms in infancy and childhood due to mtDNA depletion (5). The later onset of symptoms due to mtDNA deletions is likely due to the multiple copies of mtDNA within cells, and therefore the time taken for mtDNA deletions to reach a proportion at which a critical biochemical threshold, necessary to cause a bioenergetic deficit, is exceeded.

Clonally expanded mtDNA deletions are known to be an underlying cause of mitochondrial oxidative phosphorylation (OXPHOS) deficiency in post-mitotic cells, with studies demonstrating higher levels of mtDNA deletion heteroplasmy in cytochrome *c* oxidase (COX; complex IV)-deficient cells (6–9). These clonally expanded mtDNA deletions form and then accumulate at different times in adjacent cells leading to heterogeneity and mosaicism of adjacent muscle fibres (10). Recent work has demonstrated that the threshold level of a single mtDNA deletion required to cause OXPHOS deficiency is different for complex I and complex IV (11). Furthermore, different thresholds are observed depending on the size and location of the resulting mtDNA deletion that together determine the number of complex I (CI), CIV and tRNA genes deleted (11).

Whilst these relationships appear straightforward in patients with single, large-scale mtDNA deletions, they are likely to be more complex in patients with multiple mtDNA deletions. A systematic analysis to assess the relationship between mtDNA genetics and OXPHOS deficiency at a single cell level has not been completed for patients with multiple mtDNA deletions. Furthermore, a range of pathogenic variants in numerous Mendelian-inherited mtDNA maintenance genes can lead to the accumulation of multiple mtDNA deletions and there is no comparison of genotype differences in mtDNA deletion spectra, and OXPHOS deficiency profile, of these patients.

Here we use a quantitative quadruple immunofluorescent technique (12) to assess whether there are differences in the biochemical phenotype between different autosomal recessive and autosomal dominant nuclear gene variants resulting in multiple mtDNA deletions. Quadruple immunofluorescence in combination with fibre typing and single-cell mtDNA analysis are subsequently used to correlate mtDNA deletion level and mtDNA copy number with respiratory chain phenotype and muscle fibre type to better understand the mitochondrial genetics underpinning these diseases. Finally, we use single-molecule PCR combined with real-time PCR to measure the proportions of mtDNA molecules of up to three different lengths within single cells.

## PATIENTS AND METHODS

### Patients

Muscle biopsies of 16 patients with genetically and clinically characterized mitochondrial disease of nuclear origin were included in the study, six of them were further investigated using single-cell studies. Patients had a range of mtDNA maintenance disorders with diagnostically confirmed, segregating pathogenic variants in *POLG, TWNK, SLC25A4* and *RRM2B*.

Clinical data and genotypes are summarized in Table 1. Genetic and data analysis were performed in compliance with protocols approved by the Ethical Committee of the Martin Luther University Halle-Wittenberg. Written informed consent was obtained from all participants prior to study inclusion. Three of the cases (patients 7, 8 and 16) were investigated with informed consent by the Newcastle and North Tyneside Local Research Ethics Committees (reference 2002/205). Control tissue was acquired with prior informed consent from people undergoing anterior cruciate ligament surgery, following approval by Newcastle and North Tyneside Local Research Ethics Committees (reference 12/NE/0395).

**Table 1.** Clinical information from patients included in this study

Subjects	Gender	Age of onset	Age at biopsy	Phenotype	Skeletal muscle histochemistry	Multiple mtDNA deletions	Genotype
Patients with autosomal dominant mutations							
P 1	f	36	46	CPEO	30–40% COX-ve; 3–5% RRF	Southern blot, LRPCR	p.(Phe961Ser) ***POLG***
P 2	f	n.k.	39	CPEO	9% COX-ve; increased number of RRF	LRPCR, qPCR	p.(Tyr955Cys) ***POLG***
P 3	m	Since youth	52	CPEO, hypoacusis, neuropathy, muscle weakness, exercise intolerance	10% COX-ve; 2% RRF	Southern blot, LRPCR	p.(Lys319Glu) ***TWNK***
P 4	f	57	60	CPEO, deafness	8–10% COX-ve; 1% RRF	LRPCR	p.(Met455Thr) ***TWNK***
P 5	f	48	58	CPEO	slightly increased number of COX-ve; 1% RRF	LRPCR	p.(Arg354Pro) ***TWNK***
P 6 ^a,b^	m	n.k.	47	CPEO, bilateral ptosis	20% COX-ve; 8% RRF	LRPCR	p.(Asp104Gly) ***SLC25A4***
P7^c^	f	n.a	31	CPEO	10% COX-ve, 1% RRF	LRPCR	p.(Arg374Gln) ***TWNK***
Patients with autosomal recessive mutations							
P 8 ^d^	m	n.k.	36	Severe CPEO, ptosis, proximal muscle weakness, facial weakness, scapulae winging, low BMI, hypogonadism and osteoporosis	45% COX-ve; 15% RRF	LRPCR	p.(Thr144Ile) and p.(Gly273Ser) ***RRM2B***
P 9 ^a,c, e^	f	n.k.	43	Severe CPEO, asymmetrical ptosis, proximal and distal muscle weakness, ataxia, SNHL, facial weakness, low BMI, leukoencephalopathy and depression	22% COX-ve; 8% RRF	LRPCR	p.(Arg186Gly) and p.(Thr218Ile) ***RRM2B***
P 10	m	Since youth	54	CPEO, dementia, neuropathy	20% COX-ve; 10% RRF	Southern blot, LRPCR	p.(Gly848Sser) and p.(Ala899Thr) ***POLG***
P 11	m	23	25	Stroke-like episode, ataxia, proximal muscle weakness, dysphagia	5–7% COX-ve; no RRF	LRPCR	p.(Gly848Ser) and p.(Arg627Gln) ***POLG***
P 12	m	n.k.	43	CPEO, muscle weakness	22% COX-ve; 1% RRF;	Southern blot, LGPCR	homozygous p.(Trp748Ser) ***POLG***
P 13^f^	f	23	37	CPEO, neuropathy, dysarthria, seizures	<1% COX-ve; <1% RRF	LRPCR	p.(Arg467Thr) and p.(Trp748Ser) ***POLG***
P 14	f	67	77	CPEO, neuropathy	10% COX-ve; 1% RRF	LRPCR	p.(Arg467Thr) and p.(Thr919Leu) ***POLG***
P 15	f	26	36	CPEO	17%, COX-ve, 9% RRF	Southern blot, LRPCR	homozygous p.(Gly426Ser) ***POLG***
P 16	m	25	80	CPEO	20% COX-ve, 4% RRF	LRPCR	p.(Thr251Ile) and p.(Arg467Thr) ***POLG***

Key: y = years old; CPEO = chronic progressive external ophthalmoplegia; SNHL = Sensorineural hearing loss; BMI = body mass index; RRF = ragged red fibres; COX-ve = COX-deficient fibres; LRPCR = long range PCR; n.k = not known. a-f indicate previously published cases. a: Rocha *et al.* (2015), b: Deschauer *et al.* (2015), c: Vincent *et al.* (2018), d: Gorman *et al.* (2015), e: Pitceathly *et al.* (2012), f: Hanisch *et al.* (2015).

### OXPHOS quadruple immunofluorescence

Quadruple immunofluorescence was carried out on transverse muscle cryosections (10 μm) as described previously (12). An antibody against CI subunit NDUFB8 was used to detect CI deficiency. CIV was detected by an antibody against mtDNA encoded COX subunit I (COX-I). Porin, as an outer mitochondrial membrane voltage-gated ion channel, was used to quantify mitochondrial mass and laminin, as a basement membrane glycoprotein, was used to label myofibre boundaries. Analysis was performed as reported previously (12).

### Fibre typing

Fibre typing was carried out on transverse muscle cryosections (20 μm) from six patients. Serial sections of 10 μm were used for quadruple immunofluorescence, so the fibres could be matched after staining for OXPHOS targets. The protocol was performed as previously (13), but with some modifications according to Rocha *et al.* (11).

### Matching of fibres and grouping according to their respiratory chain deficiency

**Figure 1.**
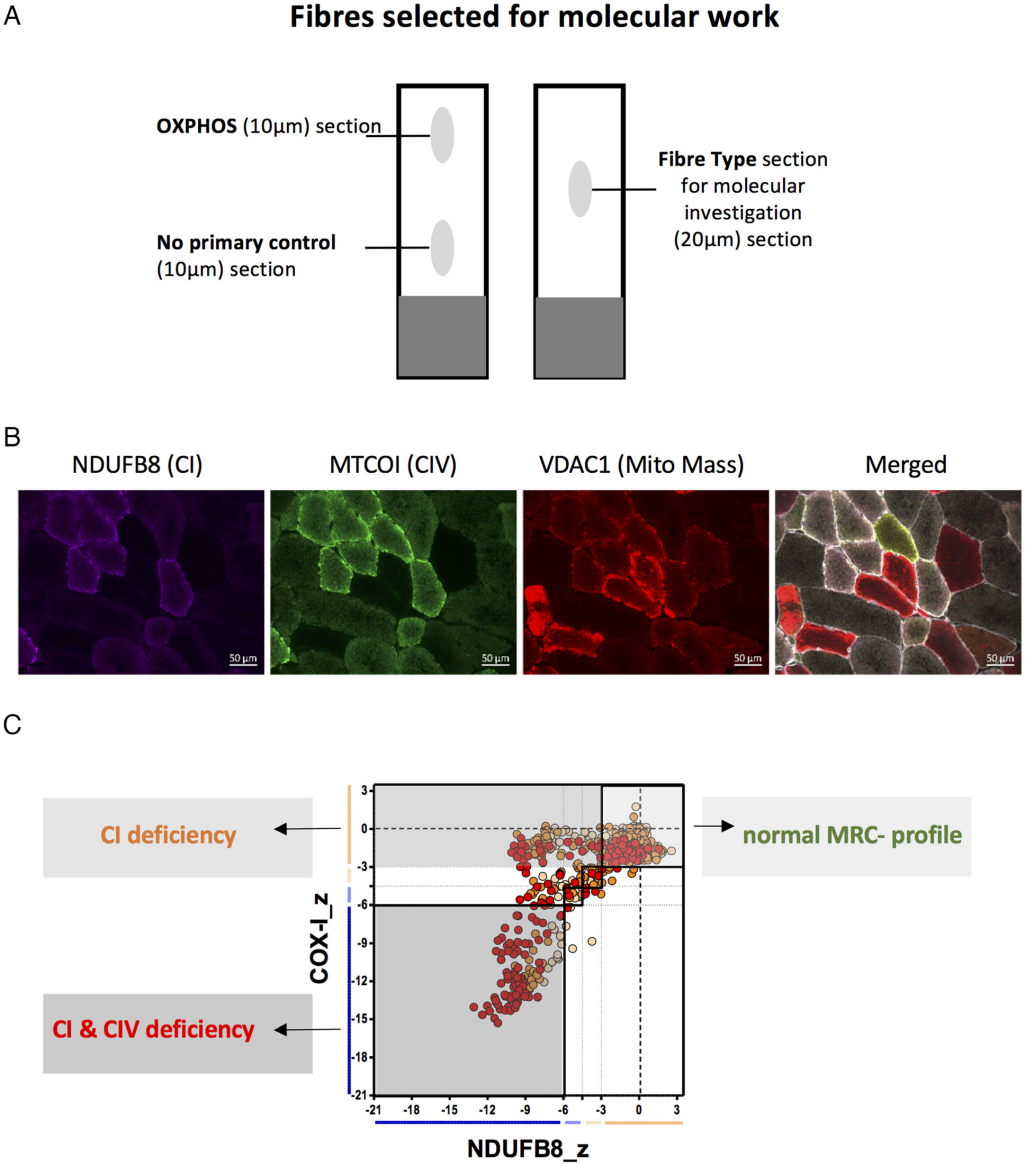
Quadruple immunofluorescence and selection of fibres for the molecular analysis of six patients. (**A**) Three serial muscle sections were taken from each patient biopsy for OXPHOS and fibre type analysis. The OXPHOS section was sequentially overlaid with all primary and secondary antibodies, whereas the no primary control section was only overlaid with the laminin antibody and all secondary antibodies. The fibre type section was overlaid with antibodies, detecting type I and type II myosin heavy chain. (**B**) Example quadruple immunofluorescent images for patient 3 with a TWNK variant. NDUFB8 is used as a complex I (CI) marker, MTCOI (COXI) as a complex IV (CIV) and VDAC1 as a mitochondrial mass marker. The merged image also includes the membrane marker laminin; scale bar: 50 μm. (**C**) Each cell of the fibre typed section was then matched with the corresponding fibre from the quadruple immunofluorescence staining. Depending of the ODporin values and the different fibre types (type 1, type 2a, type 2x), fibres were selected randomly out of each group for molecular work.

After quadruple immunofluorescence staining, fibres were grouped depending on their respiratory chain deficiency into three different groups (Figure 1). The first group were completely normal for levels of CI and CIV (CI+ and CIV+), the second CI-deficient (CI- and CIV+) and the third CI- and CIV-deficient (CI- and CIV-).

Each cell of the fibre-typed section was then matched with the corresponding fibre from the quadruple immunofluorescence staining. In each of the three MRC groups, fibres were grouped according to their ODporin value and according to their fibre type (type 1, type 2a, type 2x). Finally, fibres were selected randomly out of all groups for molecular analysis (Figure 1).

### Image acquisition

Brightfield and fluorescent images were acquired at 20× magnification using a Zeiss Axio Imager Z2 with a motorized stage and AxioVision (Release.4.8.2) and Zen 2 pro software, with monochrome (AxioCam MRm) and colour (AxioCam MRc) Digital Cameras and filter cubes for Alexa Fluor dyes at 750 nm (laminin), 488 nm (COX-I), 546 nm (porin) and 647 nm (NDUFB8) wavelengths, respectively. Exposure time setup, image record and image processing have been performed as described previously (12).

### Densitometry measurements and data analysis

Fluorescent images were analysed using an in-house developed Matlab-based software (available upon request). Exported data were uploaded to a website to perform background correction and statistical analysis (http://research.ncl.ac.uk/mitoresearch/). Fibre-surfaces were created automatically over all channels; however, unwanted areas like vessels, folds or background were removed manually. At the end of fibre analysis, the software created a merged data-file, including all individual values of patients and controls, which has been exported as a .csv for further statistical analysis according to Rocha *et al.* (12). Using the merged data, MRC-plots for each patient and control have been created. The plots included *z*-scores for COX-I, NDUFB8 and Porin values for each myofibre analysed.

### Real-time PCR analysis from single cells

A triplex real-time PCR assay was used to quantify mtDNA deletion level as described previously (14). This method quantifies relative levels of *MT-ND4*, *MT-ND1* and D-Loop, using TaqMan chemistry. Primers and TaqMan MGB probes used have been previously reported (14,15). PCR amplification was completed in a 15 μl reaction in triplicate for each sample, with each plate containing a serial dilution of p7D1 plasmid for standard curve generation, as reported previously (14). Due to double/triple-stranded nature of the D-Loop region, we considered samples to be normal when the *MT-ND1*/ D-Loop or *MT-ND4*/D-Loop percentage deletion was between 0 (all mtDNA copies contain double-stranded D-Loop) and 33% (all mtDNA copies contain triple-stranded D-Loop), as described previously (14). Therefore, values of >33% indicated the presence of an mtDNA deletion species.

### Long-range PCR

Long-range PCR was carried out using LA Taq DNA polymerase (TaKaRa) on individual myofibres. For ∼16 Kb amplification of individual myofibres primer pair 3F (m.3-27) and 16360R (m.16360-16384) were used, and for 12 Kb amplification primer pairs 2549F (m.2549-2569) and 14375R (m.14375-14356) or 323F (m.323-343) and 11605R (m.11605-11586) were used (NC_012920.1). For all long-range PCR reactions, LA Taq reaction (50 μl) was prepared as follows: 5 μl buffer, 0.4 μM each of dNTPs, 0.4 μM forward and reverse primers, 2.5 units of polymerase, 2 μl DNA and nuclease-free water up to 50 μl. Cycling conditions were: 94°C for 1 min; 35 cycles of 94°C for 30 s, 58°C for 30 s and 68°C for 16 min; 72°C for 19 min.

### mtDNA deletion breakpoint sequencing

Long-range PCR products were cleaned up using Agencourt AMPure XP beads and quantity and quality assessed with a NanoDrop (ND-1000, Labtech international). About 100 ng of each DNA sample was then fragmented, barcoded, size-selected and amplified using IonXpress Plus Fragment Library Kit, Ion Xpress Barcode Adapters and E-Gel Size Select 2% agarose gels (Thermo Fisher Scientific), according to the manufacturer’s recommendations. Individual libraries were quantified using an Agilent Bioanalyzer DNA High Sensitivity kit and pooled in equimolar concentrations.

Template preparation and Ion 316 chip v2 loading were performed on the Ion Chef (Thermo Fisher Scientific) and chips were sequenced on the Ion PGM (Thermo Fisher Scientific) using the Ion PGM Hi-Q View Chef kit as per the manufacturer’s instructions (Thermo Fisher Scientific). Data alignments to the revised Cambridge mtDNA reference sequence (NC_012920.1) were completed with the Torrent Suite software v5.4.0. Aligned data were visualized and mtDNA deletion breakpoints identified using IGV v2.3.75 (Broad Institute) (16). Sequencing data were deposited in the NCBI sequence read archive (Accession number: PRJNA527002).

### Assessment of mtDNA deletion proportions in single cells

Serial cryosections from three patients were treated for COX/SDH histochemistry (first and third section) and SDH histochemistry (second section). The SDH slide was used for laser micro-dissection of COX-deficient fibres (as assessed by COX/SDH histochemistry on adjacent sections). Single cells were laser micro-dissected (Zeiss PALM laser capture microscope) and lysed using a QIAmp DNA micro (Qiagen), following the manufacturer’s instructions. Single cell lysates were assessed using single molecule PCR and ND1/ND4 real-time assay (15).

Single-molecule PCR (smPCR) is an artefact-free approach to analyse mtDNA deletions, since amplification occurs from a single mtDNA molecule (17,18). For smPCR, the extracted DNA from above was diluted to a template concentration that yielded an amplicon from less than a quarter of PCR reactions. smPCR was carried out as two reactions, as described previously (19). For the first round, primers were 2999F (m.2999-3028) and 2949R (m.2949-2918), and PCR products from the first round were used with 3160F (m.3160-3191) and 2949R (m.2462-2494) for the second round. Based on the proportion of wells containing an amplicon, it was possible to calculate the dilution of template DNA needed to achieve single molecule PCR based on a Poisson probability table. The above two-round PCR was repeated with the desired template concentration, yielding no more than a 1 in 4 success rate across a 96-well plate. The proportion of wells containing an amplicon was used to determine the relative proportion of different mtDNA deletion species.

## RESULTS

Serial sections from skeletal muscle biopsies of patients with mtDNA maintenance disorders were subjected to immunofluorescent analyses to assess respiratory chain protein levels and fibre type (Figure 1). The set-up of the experiment allowed subsequent single-cell genetic analysis.

### Different mtDNA maintenance disorder genotypes have similar biochemical signatures

**Figure 2.**
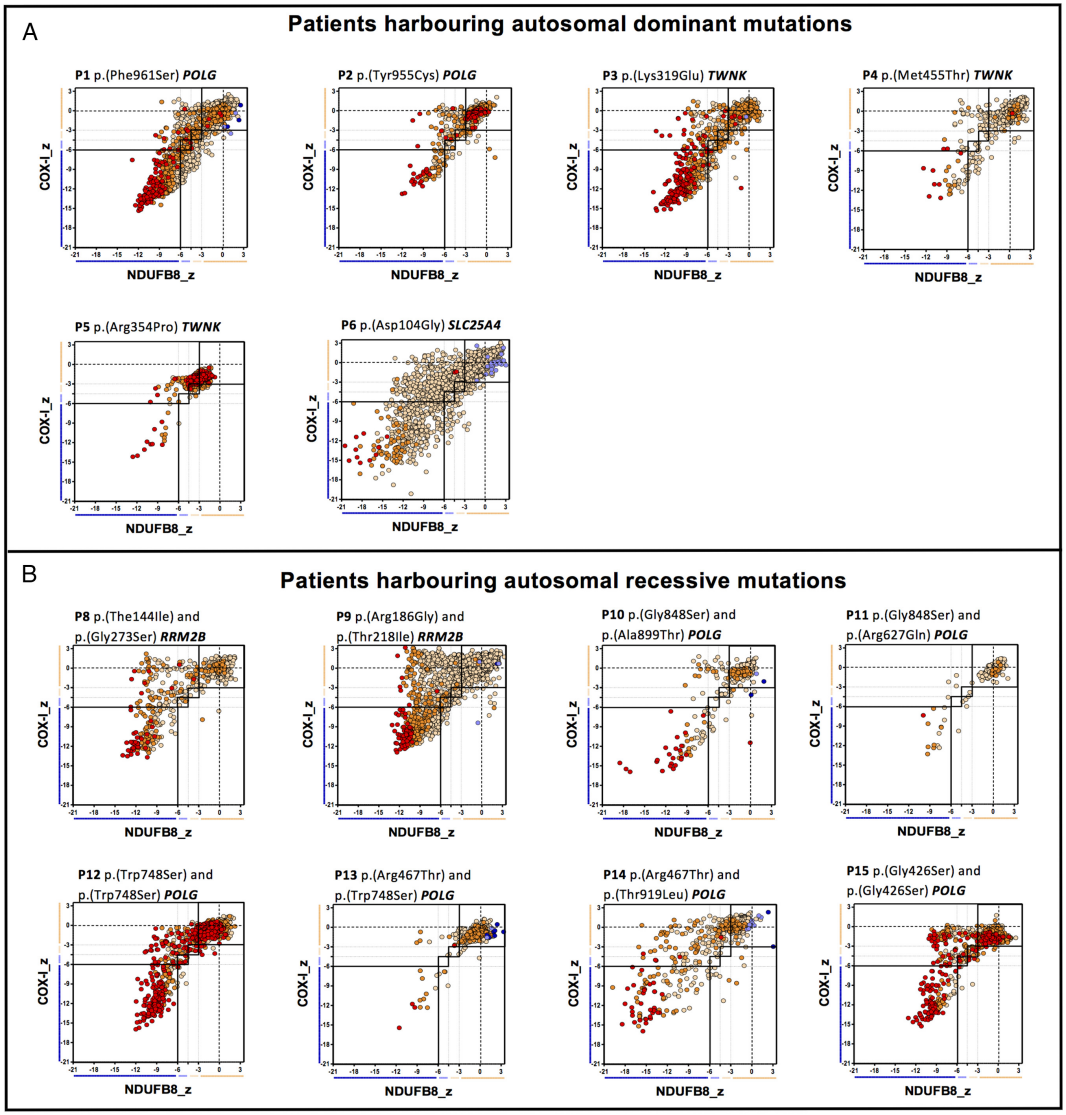
Mitochondrial respiratory chain (MRC) expression profile for complex I, complex IV and porin levels. Plots show complex I and IV expression profile from patients with (**A**) autosomal dominant mutations, *n* = 6 and (**B**) autosomal-recessive mutations, *n* = 8. Each dot represents the measurement from an individual muscle fibre, colour coded according to its mitochondrial mass (very low: blue, low: light blue, normal: light orange, high: orange and very high: red). Thin black dashed lines indicate the SD limits for the classification of fibres, lines next to *x* and *y* axis indicate the levels of NDUFB8 and COX-I, respectively (beige: normal, light beige: intermediate(+), light blue: intermediate(−) and blue: deficient). Bold dashed lines indicate the mean expression level of normal fibres. Immunofluorescent data used to generate these plots is provided in Supplementary Data file 1.

Quantitative quadruple immunofluorescent (IF) staining was used to evaluate biochemical signatures in patients with mtDNA maintenance disorders (*n* = 14) and to assess whether there is a difference between recessively and dominantly inherited pathogenic variants. Data provided in supplementary data file 1. Comparing the IF images of all patients we observed MRC-profiles similar to those previously reported (12), with no obvious differences between dominant (Figure 2A) and recessive (Figure 2B) mutations. We defined three groups of fibres: respiratory chain normal, CI-deficient or CI- and CIV-deficient (Figure 2). This pattern of deficiency was particularly obvious for those patients showing more than 20% COX-deficient fibres in muscle biopsy (Table 1), e.g. P1, P6, P7, P8, P9, P11.

Ragged-red fibres (RRF) are a common finding in patients with adult-onset mtDNA maintenance disorders and demonstrate hyperproliferation of mitochondria in a subset of muscle fibres. We used high porin levels as a marker for RRF fibres and compared cells from images of muscle fibres previously classified as normal and RRF by visual inspection. Based on the porin *z*-scores for a subset of classified fibres, we defined RRF fibres as those with a porin *z*-score > 2.5. We find that the majority (535 out of 636 or 84.1%) of RRF are deficient for both CI and CIV, 10.5% (67 out of 636) have isolated CIV deficiency and only 0.3% (2 out of 636) have isolated CI deficiency. The majority (30 out of 38 or 78.9%) of RRF are classified as type I muscle fibres, with 18.4% (7 out of 38) being type IIa and 2.6% (1 out of 38) type IIb.

### Respiratory chain deficiency is associated with a higher level of mtDNA deletion at a single-cell level

**Figure 3.**
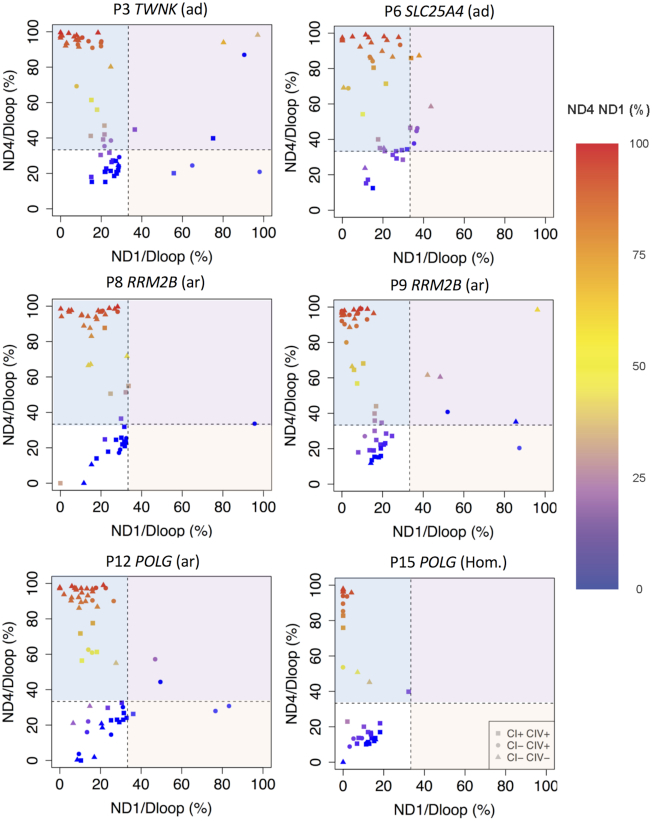
Triplex real-time PCR results of six patients. Deletions as proportions of two mtDNA targets: *MT-ND4*/*MT-ND1* is the proportion of *MT-ND4* per *MT-ND1*. *MT-ND1*/D-Loop is the proportion of *MT-ND1* relative to the D-Loop. *MT-ND4*/D-Loop is the proportion of *MT-ND4* relative to the D-Loop. Every point represents a single muscle fibre, analysed by real-time PCR. The squares show muscle fibres classed as normal for both complex I and complex IV, circles represent fibres deficient for complex I and normal for complex IV and triangles deficient for both complex I and IV. The dashed line marks where *MT-ND4*/D-loop and *MT-ND1*/D-loop percentage deletion equals 33%. Fibres ≤ 33% of *MT-ND1/*D-Loop or MT-*ND4/*D-Loop percentage deletion were considered to be without any deletion due to the double*/*triple-stranded nature of the D-Loop region. Colouring indicates the *MT-ND4*/*MT-ND1* proportion. Areas corresponding to deletions of *MT-ND4* are shaded in light blue, deletions of *MT-ND1* in light red and both *MT-ND1* and *MT-ND4* in light purple. Data used to generate these plots is provided in supplementary data file 2.

To correlate the underlying mtDNA deletions with the biochemical deficiency at a single-cell level, six patients (P3, P6, P7, P8, P11 and P14) from the 14 that underwent quadruple immunofluorescence were selected for genetic analysis by triplex real-time PCR (Figure 3). Raw Cq, mtDNA copy number and deletion level for each cell is provided in supplementary data file 2. In all six patients, we observed that mtDNA deletion level is higher in fibres with CI or CI and CIV deficiency than those which are respiratory chain normal (Figure 3). In order to retain multiplexed data from the triplex real-time assay and assess this in the context of respiratory chain deficiency, 2D scatter plots of *MT-ND1*/D-Loop versus *MT-ND4*/D-Loop were coloured based on *MT-ND4*/*MT-ND1* (Figure 3). mtDNA deletions leading to a loss of *MT-ND4* were most prevalent (85.6% of all deleted species, 166 out of 194) in all six patients (light blue box, Figure 3), with deletions encompassing *MT-ND1* but not *MT-ND4* (light orange box, Figure 3) less common (3.1%, 6 out of 194) and observed in four of six patients (P3, P7, P8 and P11). Finally, four out of six patients also had deletions encompassing both *MT-ND1* and *MT-ND4* (11.3%, 22 out of 194) (light purple box, Figure 3, P3, P6, P8 and P11).

### Respiratory chain deficiency is not associated with mtDNA depletion in these patients

**Figure 4.**
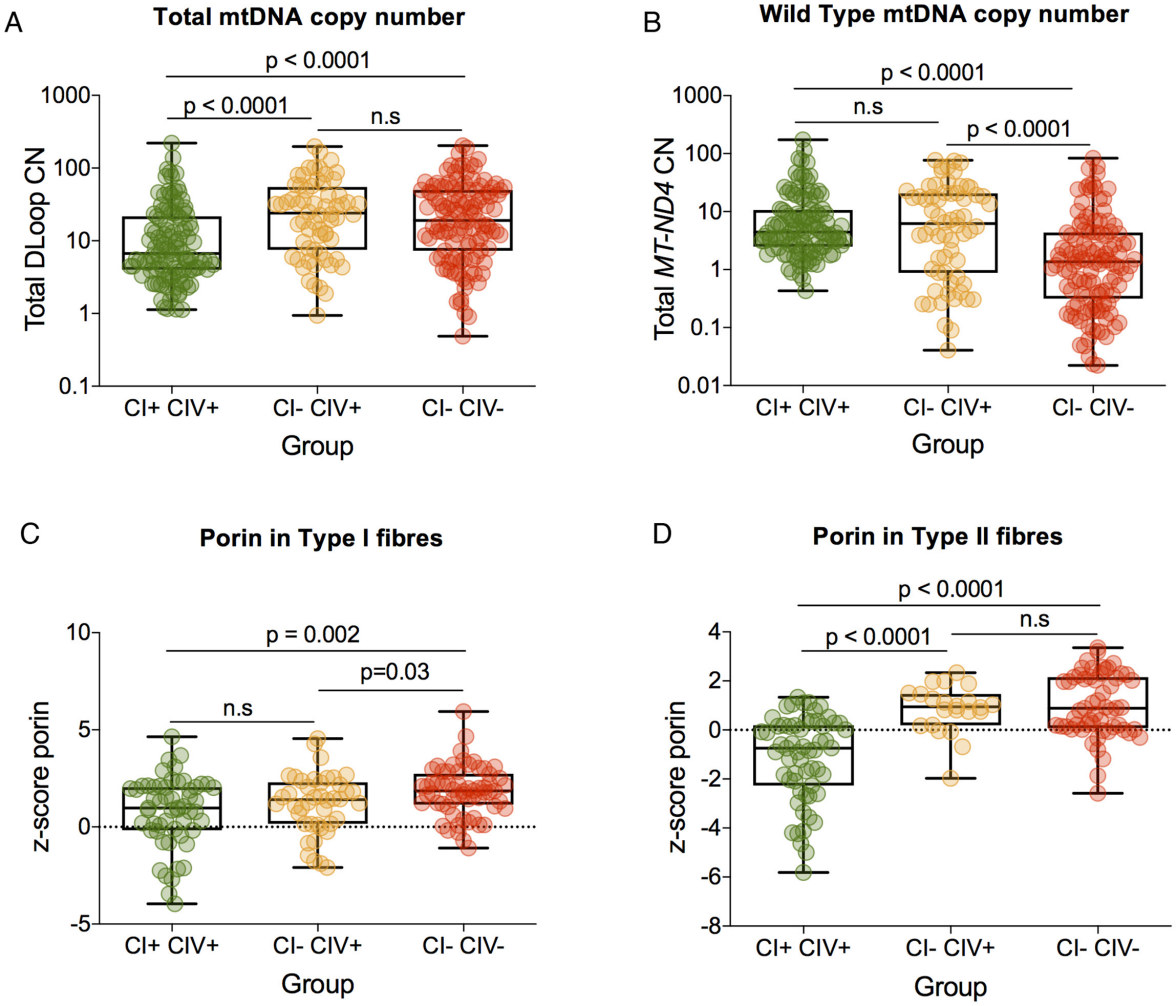
Exploring relationship between mtDNA copy number and mitochondrial mass with respiratory chain deficiency in patients 3, 7–10 and 16. For all plots box and whiskers show mean, 25th and 75th percentiles, minimum and maximum. (**A**) D-Loop (total) mtDNA copy number fibres were grouped as CI+ CIV+, CI- CIV+ and CI- CIV-. A significantly higher level of copy number is detected in both CI- CIV+ and CI- CIV- compared to CI+ CIV+ (*P* < 0.0001, Mann–Whitney). No significant difference is noted between CI- CIV+ and CI- CIV- (Mann–Whitney). (**B**) ND4 (wild-type) mtDNA copy number for CI+ CIV+, CI- CIV+ and CI CIV- fibres. No significant difference in copy mtDNA copy number between CI+ CIV+ and group CI- CIV+ (Mann–Whitney). In comparison, there is a significantly lower wild-type copy number in groups CI- CIV- compared to groups CI+ CIV+ and CI- CIV+ (*P* < 0.0001, Mann–Whitney). (**C**) Porin level for CI + CIV+, CI- CIV+ and CI- CIV- type I muscle fibres. (**D**) Porin level for CI+ CIV+, CI- CIV+, CI- CIV- type II fibres.

Respiratory chain deficiency can also be caused by a depletion of mitochondrial mass and mtDNA. Therefore, we sought to investigate whether respiratory chain deficiency was associated with a quantitative loss of mtDNA copy number and mitochondrial mass (Figure 4). Comparing all three groups of fibres, no obvious sign of mtDNA depletion was observed in respiratory-deficient muscle fibres compared to normal muscle fibres. In contrast, a significantly higher level of total D-Loop mtDNA copy number was detected in fibres with CI deficiency or with both CI and CIV deficiency, compared to normal fibres (*P* < 0.0001, A; *n* = 114, B; *n* = 64, C; *n* = 118). However, no significant difference was detected between CI-deficient and CI- and CIV-deficient fibres (Figure 4A).

Since a reduction in wild-type copy number is associated with respiratory chain deficiency, we examined the relationship between *MT-ND4* as a measure of wild-type copy number and respiratory chain deficiency (Figure 4B). *MT-ND4* wild-type mtDNA copy number showed no significant difference between normal fibres (*n* = 114) and CI-deficient fibres (*n* = 64). Wild-type mtDNA copy number was significantly lower in CI- and CIV-deficient fibres (*P* < 0.0001, *n* = 118, Figure 4B).

Since a change in total copy number may also be associated with a change in mitochondrial mass, we looked to see whether there were significant differences between porin levels in Type I (Figure 4C) and type II (Figure 4D) fibres. We find that mitochondrial mass is significantly higher in CI- and CIV-deficient fibres than normal or CI-deficient fibres in type I fibres. In type II fibres, mitochondrial mass is significantly higher in both CI- deficient and CI- and CIV-deficient fibres than CI normal fibres.

### We see up to three mtDNA deletion species in a single muscle fibre

**Figure 5.**
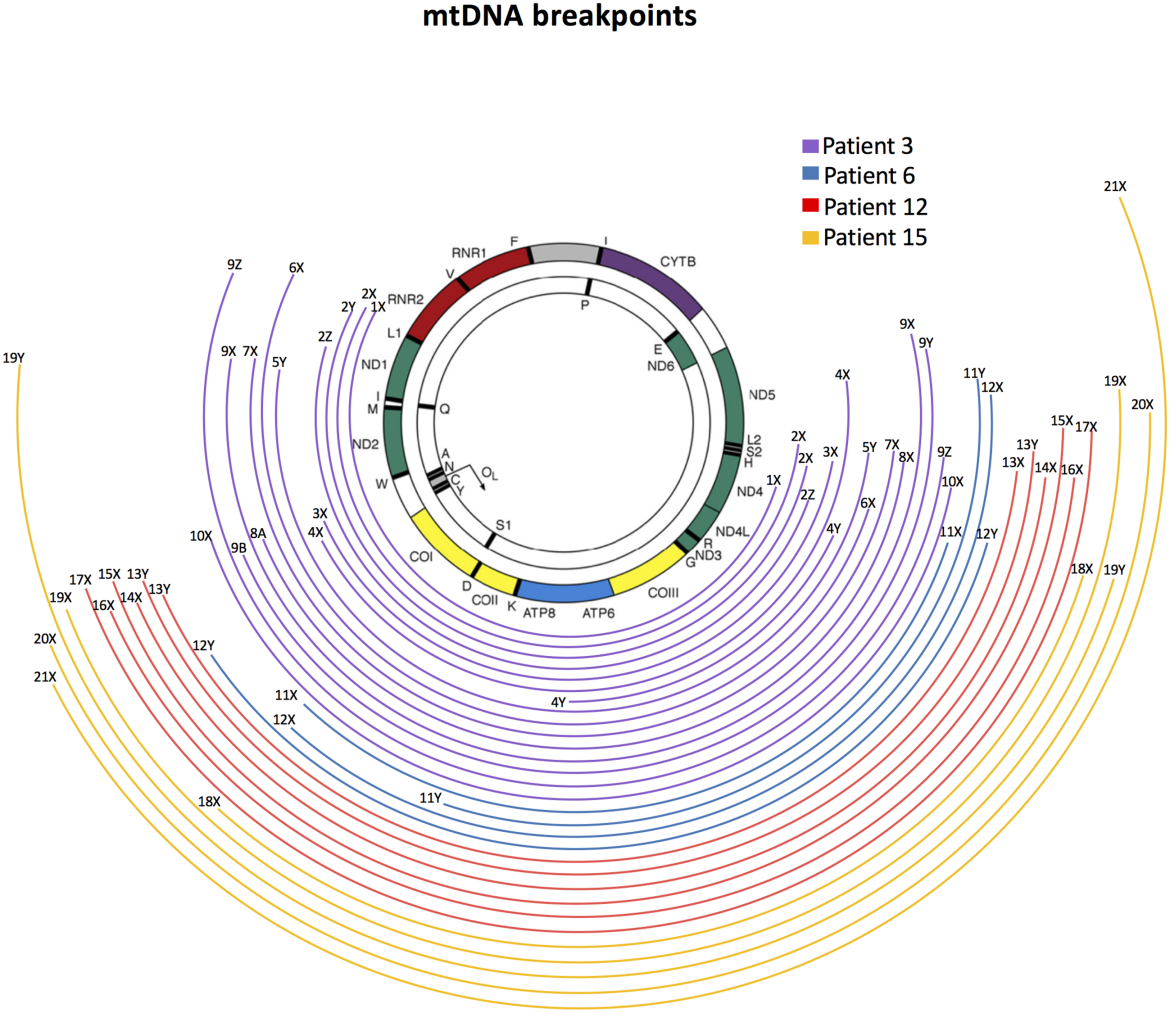
Mitochondrial DNA deletion spectra from patients 3, 6, 12 and 15. Long range PCR was used to amplify the mitochondrial DNA (mtDNA) from single muscle fibres (*n* = 21), and then the amplicons sequenced to determine breakpoints. Size and position within the mitochondrial genome of all mtDNA deletions identified by 12 Kb long-range PCR are presented as coloured curved lines. Lines endings on the left and right mark the 5′ and 3’ breakpoints, respectively.

Since real-time PCR does not indicate how many mtDNA deletions or what size mtDNA deletions are contained within each cell, we selected a subset of cells and amplified mtDNA by long range PCR to detect mtDNA deletions. DNA from 21 single muscle fibres from patients 3, 6, 7 and 14 was amplified, and the mtDNA breakpoints sequenced (Figure 5). In Figure 5, mtDNA sequences are labelled with a cell number (1-21) and a letter (X,Y,Z) denoting unique mtDNA sequence (deletion species) in a single cell. Of these 21 cells, 66.7% (14 cells) harboured a single mtDNA deletion, 23.8% (5 cells) had two detectable mtDNA deletions and 10.0% (2 cells) harboured three mtDNA deletions (Table 2) with a total of 31 mtDNA deletion species identified overall. All mtDNA deletions removed at least part of the major arc with half of them also removing part of the minor arc (Figure 5). Most deletions removing both *MT-ND1* and *MT-ND4* are from P3 (*n* = 9), with only one from P14. For all fibres where both breakpoint sequencing and real-time data were available, the two methods provided agreement on the removal of *MT-ND1, MT-ND4* or both. Of 31 mtDNA deletions sequenced 13 (42%) had a repeat sequence of 2–11 bp in length at the break-point, of these all were perfect repeats.

**Table 2.** Characterization of mtDNA deletions found by long-range PCR and breakpoint sequencing. If breakpoints contained a repeat, only those with a repeat 3 bp or longer were indicated; any repeats 2 bp or shorter were classed as no repeat

PCR Product	Patient	np 5' breakpoint	np 3' breakpoint	Primers used	Deletion size based on sequencing [bp]	ND1/D-Loop [%]	ND4/D-Loop [%]	5′ breakpoint sequence	3′ breakpoint sequence	Repeats at breakpoints
1X	P3	3270	11195	323F/12054R	7924	75.1	39.8	5′-GGTAATCGCATAAAACTTAAAAC-3′	5′-GGCACATACTTCCTATT-3′	no repeat
2X	P3	3263	12299	2549F/14374R	9035	90.5	86.9	5′-TAATCGCATAAAAC-3′	5′-AGGCCCCAAAAATTTT-3′	no repeat
2Y		4351	13924		9572			5′-TATGAGAATCGAACCC-3′	5′CCCTAGCATCACACACC-3′	no repeat
2Z		3326	11668		8341			5′-CATACCCATGGCCAACs**(CTCCT)**-3′	5′-**(CTCCT)**CCAAACCCCC-3′	5bp perfect repeat
3X	P3	5791	12302		6510	19.9	93.8	5′-GGCAGGTTTGAAGCTGCTTCTTCG-3′	5′-GCCCCAAAAATTTTGGT-3′	no repeat
4X	P3	5786	13238		7451	7.9	69.3	5′-CAGGTTTGAAGCTGCTT-3′	5′-TCGTAGCCTTCTCCAC-3′	no repeat
4Y		8591	11464		2872			5′-GGCCTACCC**(GCCGCAGTACT)**-3′	5′-**(GCCGCAGTACT)**CTTAAAACTAGGC-3′	11bp perfect repeat
5X	P3	3914	12299		8384	19.9	91.9	5′-CCCCCTTCGACCTTGCCGAAGG-3′	5′-AGGCCCCAAAAATTTT-3′	no repeat
6X	P3	3263	11493	323F/12054R	8229	24.7	80.2	5′-TAATCGC**(ATAAAAC)**-3′	5′-**(ATAAAAC)**GCCTCACACTCATT-3′	7bp perfect repeat
7X	P3	4351	12111	2549F/14374R	7759	4.0	99.1	5′-TAGGACTATGAGAATCGAACCC-3′	5′-CCCCGACATCATTACCGG-3′	no repeat
8X	P3	5787	13924		8136	7.5	95.4	5′-CAGGTTTGAAGCTGCTTC-3′	5′CCCTAGCATCACACACCG-3′	no repeat
9X	P3	4376	13923		9546	18.4	99.5	5′-GAATCCAAAATTCTCCG-3′	5′-CCCTAGCATCACACACCG-3′	no repeat
9Y		5786	13238		7451			5′-CAGGTTTGAAGCTGCTT-3′	5′-TCGTAGCCTTCTCCAC-3′	no repeat
9Z		3260	12299		9038			5′-GCCCGGTAATCGCATAA-3′	5′-AGGCCCCAAAAATTTTGGT-3′	no repeat
10X	P3	5825	13923		8097	0.0	96.6	5′-AAAATCACCTCGGAGC-3′	5′-CCCTAGCATCACACACC-3′	no repeat
11X	P6	6226	12107	2549F/14374R	5880	14.2	94.6	5′TTCTGACTCTTACCCC**(TCCCTC)**-3′	5′-**(TCCCTC)**AACCCCGAC-3′	6bp perfect repeat
11Y		8482	13460		4977			5′-CCACCT**(ACCTCCCTCACCA)**-3′	5′-**(ACCTCCCTCACCA)**TTGGCAGCCTAGCA-3′	13bp perfect repeat
12X	P6	7809	14001		6191	8.8	92.3	5′TCCTGCCCGCCATCA**(TCCTAGACCT)**-3′	5′-**(TCCTAGACCT)**AACCTGACTAGAAAA-3′	10bp perfect repeat
12Y		6226	12107		5880			5′-TTCTGACTCTTACCCC**(TCCCTC)**-3′	5′-**(TCCCTC)**AACCCCGAC-3′	6bp perfect repeat
13X	P11	6341	14005		7663	95.6	33.7	5′-GCCTCCG**(TAGACCTAACC)**-3′	5′-**(TAGACCTAACC)**TGACTAGAAAAGCTA-3′	11bp perfect repeat
13Y		5787	13923	2549F/14374R	8135			5′-CAGGTTTGAAGCTGCTTC-3′	5′-CCCTAGCATCACACACCG-3′	no repeat
14X	P11	6880	13457		6576	18.6	97.4	5′-TTTAGCTGACTCGCCAC-3′	5′-CCATTGGCAGCCTAGCATT-3′	no repeat
15X	P11	5787	13924		8136	14.9	67.2	5′-CAGGTTTGAAGCTGCTTC-3′	5′-CCTAGCATCACACACCG-3′	no repeat
16X	P11	6341	14005		7663	13.6	94.2	5′-GCCTCCG**(TAGACCTAACC)**-3′	5′-**(TAGACCTAACC)**TGACTAGAAAAGCTA-3′	11bp perfect repeat
17X	P11	5788	13922		8133	10.3	94.6	5′-CAGGTTTGAAGCTGC**(TTCT)**-3′	5′-**(TTCT)**ACCCTAGCATCACACACC-3′	4bp perfect repeat
18X	P14	3270	11195	2549F/14374R	7924	0.0	82.8	5′-GGTAATCGCATAAAACTTAAAAC-3′	5′-GGCACATACTTCCTATT-3′	no repeat
19X	P14	5788	13923		8134	0.0	85.3	5′-GGCAGGTTTGAAGCTGCTTCT-3′	5′-CCCTAGCATCACACACC-3′	no repeat
19Y		4351	12111		7759			5′-TAGGACTATGAGAATCGAACCC-3′	5′-CCCCGACATCATTACCGG-3′	no repeat
20X	P14	5787	13923		8135	13.0	45.1	5′-CAGGTTTGAAGCTGCTTC-3′	5′-CCCTAGCATCACACACCG-3′	no repeat
21X	P14	6798	14269		7470	4.1	95.7	5′-TTTACAGTAGGAATAGACG-3′	5′-CGAATCAACCCTGACCCCTCT-3′	no repeat

### Molecular genetics do not strictly correlate with respiratory chain deficiency in fibres with multiple mtDNA deletions

Using data from quadruple immunofluorescence, real-time PCR and mtDNA deletion breakpoint sequencing we sought to correlate the molecular findings with the observed respiratory chain deficiency. However, when multiple mtDNA deletions were present in a single cell it was often not possible to tell what proportion of each mtDNA deletion species was present (Table 2). Furthermore, in contrast to the clear-cut genetic and biochemical correlations reported by Rocha *et al.* (11), where a single mtDNA deletion was identified by long range PCR, we observed some muscle fibres where all three COX genes are deleted at high levels and yet the cell demonstrates only isolated CI deficiency at the immunohistochemical level (e.g. cells 2–8, 13 and 19 in Figure 5 and Table 2).

### Data do not conclusively suggest a replicative advantage for larger mtDNA deletion species

It has previously been suggested that mtDNA deletions may clonally expand due to a replicative advantage, since deleted species are smaller and can therefore be replicated faster (20). Long-range PCR has an inherent bias towards smaller mtDNA molecules and therefore does not allow us to accurately assess the relative proportions of each mtDNA deletion. In comparison smPCR requires the dilution of template DNA down to the point that any reaction where the amplicon produced must contain only a single mtDNA molecule therefore removing this bias and allowing quantitative assessment (17). To investigate the possibility of a replicative advantage for smaller species, we performed single molecule PCR (smPCR). Single muscle fibres were laser micro-dissected from patients P7, P8 and P16 with *POLG* (*n* = 1), *TWNK* (*n* = 1) and *RRM2B* (*n* = 1) mutations respectively, and smPCR performed. The data from this are summarized in Table 3.

**Table 3.** Summary of results from single molecule PCR

			Deletion 1		Deletion 2		Deletion 3		
Fibre	No. of deletions	ND4/ND1 % deletion load	Size (Kb)	Proportion	Size (Kb)	Proportion	Size (Kb)	Proportion	Is the largest deletion highest level?
**Patient 7 *TWNK***									
1	2	72	4	0.23	5	0.77			Yes
2	1	32	5	1					
3	2	73	6	0.69	7	0.31			No
4	2	85	5	0.86	7	0.14			No
5	1	69	7	1					
6	1	29	5	1					
7	2	84	4	0.28	5	0.72			Yes
8	2	98	5	0.25	7	0.75			Yes
9	1	83	7	1					
10	1	u.m	5	1					
11	1	28	5	1					
12	1	83	7	1					
13	2	82	6	0.42	7	0.58			Yes
14	1	90	5	1					
15	1	30	6	1					
16	1	43	7	1					
17	1	81	8	1					
18	1	u.m	6	1					
19	2	47	5	0.67	10	0.33			No
20	1	86	6	1					
21	1	81	5	1					
22	1	95	5	1					
23	2	95	6	0.8	9	0.2			No
24	1	89	5	1					
25	2	61	6	0.79	10	0.21			No
26	2	77	6	0.86	10	0.14			No
**Patient 8 *RRM2B***									
1	2	17	4	0.6	5	0.4			No
2	2	67	8	0.2	9	0.8			Yes
3	1	<5	5	1					
4	1	<5	4	1					
5	1	37	5	1					
6	1	59	3	1					
7	3	97	6	0.49	6	0.4753	10	0.39	No
8	1	62	6	1					
9	1	96	5	1					
10	1	98	8	1					
11	2	37	5	0.33	6	0.67			Yes
12	1	96	7	1					
13	1	80	7	1					
14	1	37	5	1					
15	2	59	4	0.57	6	0.43			No
16	1	low level	4	1					
17	2	61	5	0.75	7	0.25			No
18	2	58	4	0.47	8	0.53			Yes
19	1	46	5	1					
20	1	70	10	1					
21	3	98	5	0.29	7	0.44	8	0.27	Yes
22	1	96	8	1					
23	2	80	4	0.27	6	0.73			Yes
24	2	96	5	0.23	7	0.77			Yes
25	1	98	4	1					
26	2	98	6	0.51	7	0.49			No
27	2	99	5	0.54	6	0.46			No
**Patient16 *POLG***									
1	1	14	4	1					
2	1	84	6	1					
3	1	91	7	1					
4	1	u.m	7	1					
5	3	82	4	0.39	5	0.23	8	0.38	No
6	1	95	5	1					
7	2	62	6	0.73	7	0.27			No
8	1	94	6	1					
9	2	67	6	0.37	7	0.63			Yes
10	1	92	7.5	1					
11	2	57	6	0.17	8.5	0.83			Yes
12	1	43	6	1					
13	2	38	6.5	0.43	7.5	0.57			Yes
14	1	89	6	1					
15	1	u.m	2	1					
16	2	23	5	0.53	7	0.47			No
17	1	46	9	1					
18	1	72	7	1					
19	1	34	7	1					
20	2	67	3	0.54	7	0.46			No
21	1	82	6	1					
22	1	31	4	1					
23	2	76	6	0.57	8	0.43			No
24	1	44	6	1					
25	1	52	7	1					
26	1	27	5	1					
Key: u.m = unmeasurable									

We find that 62.8% of affected cells contained a single mtDNA deletion, 34.6% contained two mtDNA deletion species and 2.6% three mtDNA deletion species. These values are similar to those we report above from our long-range PCR analysis and also to those previously reported in Inclusion Body Myositis (IBM) (19). We also note that there is no difference in the size of mtDNA deletions between the three different genotypes.

In the cells containing multiple mtDNA deletion species, we were able to examine whether the smaller of the species is most prevalent. We find that in P7 the largest mtDNA deletion (the smallest species of mtDNA molecule) was most prevalent in 4/10 cells (40.0%) with multiple deletions. In P8, the smallest species was most prevalent in 5/12 cells (41.7%), and in P16 the smallest mtDNA species was the most prevalent in 3/8 cells (37.5%).

## DISCUSSION

Here, we perform a single-cell investigation of the genetic and biochemical correlations and underlying pathomechanisms of patients with adult-onset mtDNA maintenance disorders. Rocha *et al.* (2018) have previously shown a strong correlation between mitochondrial genetics and respiratory chain deficiency in patients with single, large-scale mtDNA deletions. Therefore, the first aim of this study was to investigate whether a similar correlation was observed in patients with nuclear variants causing multiple mtDNA deletions. A replicative advantage of deleted—and therefore smaller—mtDNA molecules is one possible explanation for the clonal expansion of mtDNA mutations. Consequently, a second aim of this study was to ask whether a replicative advantage for smaller mtDNA molecules exists.

Quadruple immunofluorescent staining confirmed the distinct MRC-pattern previously reported in a small number of patients (12) with fibres divided into three groups: respiratory chain normal fibres, isolated CI deficiency, and cells with combined CI and CIV deficiency. We observed no obvious differences between patterns associated with recessively inherited or dominantly inherited variants, or between the different nuclear encoded mtDNA maintenance genes. This is most likely due to the stochastic nature of mtDNA deletion formation and clonal expansion in patient muscle.

We then probed deeper at a single-cell level to assess mtDNA deletion level and determine if this correlated with CI- and CIV-respiratory chain deficiency. Muscle biopsies from six patients were selected for further investigation by triplex real-time PCR in single muscle fibres. Our data showed that a higher mtDNA deletion level is associated with an increase in CI and CIV deficiency and an increase in mitochondrial mass. However, compared to results by Rocha *et al.* (11), the correlation between mitochondrial genetics and respiratory chain deficiency in mtDNA maintenance disorders is much more complicated. This is likely due to the complex nature of multiple mtDNA deletions, where patient muscle fibres sometimes contained more than one single mtDNA deletion species, which may lead to the loss of different mtDNA-encoded genes. This means that in order to build a complete picture it is also necessary to assess how many mtDNA deletions are present and where in the mtDNA these deletions are located (11).

In order to determine the number, location and proportion of mtDNA deletions present in each muscle fibre we selected a subset of fibres for long-range PCR and mtDNA sequencing. We had hoped that this would demonstrate a similar pattern in biochemical threshold as reported by Rocha *et al.* (11); however, this was not simple to determine. Of the sequenced mtDNA deletions, 10 out of 31 (36.6%) completely removed O_L_. This is similar to previous reports in IBM where sequencing demonstrated that 1 in 3 mtDNA deletions extended into the minor arc removing O_L_ (19). The existence and clonal expansion of mtDNA lacking O_L_ is difficult to explain, given previous findings that the origins of replication are essential for mtDNA replication (21). However, it has previously been demonstrated that PCR amplicons lacking O_L_ are likely to have duplications and contain both a wild-type genome as well as a deleted genome (22). Such duplications have previously been observed in IBM (19) and also in patients with MGME1 mutations (23). Unfortunately, we were unable to check for mtDNA duplications here and due to the inherent bias of long-range PCR for smaller mtDNA species we would not detect these. The presence of duplications could explain the phenotype in cells, where COXI-III genes were deleted but CIV protein levels were preserved. Of these cells, five out of nine have mtDNA deletions removing O_L_ and breakpoints that disrupt either an *MT-ND* gene or mt-tRNA. This suggests there may be duplications with breakpoints that affect CI, explaining their isolated CI deficiency, or alternatively that there are additional deletions that we are not detecting.

Deletions arise in the mtDNA of post-mitotic cells in patients with mtDNA maintenance disorders, but also during ageing, IBM and Parkinson’s disease among other disease conditions. mtDNA deletions are thought to form due to errors during replication (24), repair (25) or a combination of the two (26), with the rate of mtDNA deletion formation likely dependent on the disease, the tissue and mechanism of mtDNA replication. The sequences surrounding the breakpoints can give us insight into how these deletions were formed (25). The proportion of mtDNA deletions with repeat sequences and length of repeats we report here are similar to those reported in IBM, where 39.4% were reported to have repeats (19). In comparison in ageing muscle 80% of mtDNA deletions were found to have repeats (7), which is similar to findings in the substantia nigra in ageing, Parkinson’s disease and a patient with a *POLG* mutation (27). Whilst evidence at present points towards mtDNA deletion formation during replication (28,29), a clear mechanism for how mtDNA deletions without repeats would form during replication is missing and so their formation during repair cannot be ruled out.

Data presented here from both long-range PCR and smPCR in patients with mtDNA maintenance disorders demonstrate that multiple mtDNA deletions occur in ∼35% of muscle fibres, with up to 10% of all muscle fibres containing three mtDNA deletion species. This is similar to reports in IBM (19) and demonstrates that up to three mtDNA deletion species can clonally expand to detectable levels within a muscle fibre. There is still uncertainty as regards the exact mechanisms by which mtDNA deletions clonally expand in patients with mtDNA maintenance disorders. The random genetic drift theory (30) proposes that mtDNA mutations accumulate randomly through relaxed replication of mtDNA. ‘Survival of the smallest’ theory (20) proposes that the smaller size of mtDNA molecules containing deletions allows for faster replication and therefore gives the deleted species a replicative advantage. As such we investigated the possibility of a replicative advantage using smPCR.

If a replicative advantage for smaller mtDNA molecules were present, we would expect to see significant numbers of muscle fibres where the smallest mtDNA molecules (the largest deletions) had clonally expanded to be most prevalent. We observed the smallest species being most prevalent in ∼40% of the cells with multiple deletions that we observed across three patients. However, we cannot currently assess whether the full set of observations from these cells, as presented in Table 3, is significantly different from that we might expect under the hypothesis of random genetic drift. Assessment is complicated by the fact that a small replicative advantage might take a long time to manifest and by the fact that our patients (and indeed individual cells within patients) have experienced varying times since deletions arose. Further work is therefore required to test whether these novel, single cell data are compatible with models of clonal expansion, including survival of the smallest, random genetic drift (30) and the recently described perinuclear niche hypothesis (31).

Here we perform in-depth, single-cell analysis to attempt to correlate quantitative analysis of both mitochondrial genetics and respiratory chain deficiency in mtDNA maintenance disorders. Whilst we are able to correlate the overall mutation load with the degree of respiratory chain deficiency, the multiple mtDNA deletions make more in depth analysis of genes affected more complex. Long-range PCR and sequencing analysis do, however, demonstrate that the number of mtDNA deletions present and the percentage of breakpoints associated with repeat sequences are consistent with results from IBM. Finally, we present smPCR data that could, with further analysis, be used to assess quantitative hypotheses about the clonal expansion of mtDNA deletions.

## DATA AVAILABILITY

Sequencing data was deposited in the NCBI sequence read archive (Accession number: PRJNA527002).

## Supplementary Material

gkz472_Supplemental_Files
